# Potential of gene drives with genome editing to increase genetic gain in livestock breeding programs

**DOI:** 10.1186/s12711-016-0280-3

**Published:** 2017-01-04

**Authors:** Serap Gonen, Janez Jenko, Gregor Gorjanc, Alan J. Mileham, C. Bruce A. Whitelaw, John M. Hickey

**Affiliations:** 1 The Roslin Institute and Royal (Dick) School of Veterinary Studies, The University of Edinburgh, Easter Bush, Midlothian, Scotland, UK; 2 Genus plc, 1525 River Road, DeForest, WI 53532 USA

## Abstract

**Background:**

This paper uses simulation to explore how gene drives can increase genetic gain in livestock breeding programs. Gene drives are naturally occurring phenomena that cause a mutation on one chromosome to copy itself onto its homologous chromosome.

**Methods:**

We simulated nine different breeding and editing scenarios with a common overall structure. Each scenario began with 21 generations of selection, followed by 20 generations of selection based on true breeding values where the breeder used selection alone, selection in combination with genome editing, or selection with genome editing and gene drives. In the scenarios that used gene drives, we varied the probability of successfully incorporating the gene drive. For each scenario, we evaluated genetic gain, genetic variance $(\sigma_{A}^{2} )$, rate of change in inbreeding ($\Delta F$), number of distinct quantitative trait nucleotides (QTN) edited, rate of increase in favourable allele frequencies of edited QTN and the time to fix favourable alleles.

**Results:**

Gene drives enhanced the benefits of genome editing in seven ways: (1) they amplified the increase in genetic gain brought about by genome editing; (2) they amplified the rate of increase in the frequency of favourable alleles and reduced the time it took to fix them; (3) they enabled more rapid targeting of QTN with lesser effect for genome editing; (4) they distributed fixed editing resources across a larger number of distinct QTN across generations; (5) they focussed editing on a smaller number of QTN within a given generation; (6) they reduced the level of inbreeding when editing a subset of the sires; and (7) they increased the efficiency of converting genetic variation into genetic gain.

**Conclusions:**

Genome editing in livestock breeding results in short-, medium- and long-term increases in genetic gain. The increase in genetic gain occurs because editing increases the frequency of favourable alleles in the population. Gene drives accelerate the increase in allele frequency caused by editing, which results in even higher genetic gain over a shorter period of time with no impact on inbreeding.

**Electronic supplementary material:**

The online version of this article (doi:10.1186/s12711-016-0280-3) contains supplementary material, which is available to authorized users.

## Background

This paper uses simulation to explore how gene drives increase genetic gain in livestock breeding programs. Genetic gain is brought about by increasing the frequency of favourable alleles. In most breeding programs, the increase in frequency is achieved slowly by selecting high merit individuals as the parents of the next generation based on phenotype and/or genotype information. The efficacy and efficiency of this type of breeding program depends on four factors: the ability to accurately identify high merit individuals, the intensity of selection, the time taken to replace one generation with another and the way in which the existing genetic diversity is maintained and converted into short- and long-term genetic gain.

Recent advances in genome editing have increased interest in using this technology to accelerate genetic gain in breeding programs [1]. Genome editing allows the precise deletion, addition or change of alleles at specific locations in the genome of a cell. These changes are permanent and heritable if they are made in zygotes or germline cells.

There are over 300 examples of the use of genome editing in plants and livestock [2], including edits for herbicide resistance in oilseed rape [3], in the *myostatin* gene for “double muscling” in pigs, cattle and sheep [4], the introduction of the polled gene into dairy cattle [5], and edits to confer resistance to porcine reproductive and respiratory syndrome virus (PRRS) and African swine fever virus (ASFV) in pigs [4, 6–8].

To date, all applications of genome editing in livestock used single edits to address simple traits that are controlled by a small number of causal variants with large effects. However, in livestock breeding programs, the majority of traits of interest are quantitative and are likely affected by thousands of causal variants, each with small effect. However, although there are many causal variants for each trait, a recent simulation study using an editing strategy called PAGE (promotion of alleles by genome editing) showed that discovering and editing relatively small numbers of causal variants can double the rate of both short- and long-term genetic gain compared to selection alone [1].

Although the increase in genetic gain from PAGE was impressive, many generations of editing were needed to fix favourable alleles [1]. This is because unfavourable alleles continue to segregate within the non-edited parents (i.e., dams) of each generation. Methods that can fix favourable alleles more quickly would be valuable within breeding programs. One such method is genome editing with gene drives.

Gene drives are naturally occurring phenomena that cause a mutation on one chromosome to copy itself onto its homologous chromosome. The copying process occurs because the gene drive initiates a double-stranded DNA break on the homologous chromosome. The DNA break is repaired by cellular pathways such as homology-directed repair, which uses the sequence of the chromosome that contains the gene drive elements as a repair template [9, 10]. An example of a naturally occurring gene drive is the so-called P-element, which invaded the fruit fly *Drosophila melanogaster* in the 1950s and has since spread worldwide [11].

With advances in genome editing technology, a gene drive can be incorporated with a genome edit made either on the germline cell of a parent or on the parent itself at the zygote stage to ensure that all offspring are homozygous for the edited allele. The possibility of using gene drives to promote the spread of alleles through a population was first proposed by Burt in 2003 [12]. This concept, now recently termed the ‘mutagenic chain reaction’, was empirically demonstrated in *Drosophila* through modification of the CRISPR/Cas9 system originally identified in bacteria [13–15]. In this case, the CRISPR/Cas9 gene drive system was used to induce a change in the *Drosophila* body colour from wild type to yellow by copying the gene drive-linked *yellow* gene onto the homologous chromosome of offspring inheriting one copy of the *yellow* gene [15].

Since this demonstration, artificially constructed gene drives have gained renewed interest as a way of quickly spreading alleles in natural populations [12]. Targeted gene drive mechanisms based on CRISPR/Cas9 editors have been reported to have conversion efficacies of more than 98% [16], demonstrating the potential of this technology in spreading alleles in populations. One recent proposal is to use gene drives to spread a deleterious allele in populations of mosquito hosts of the malaria parasite. The deleterious allele reduces the fitness of the mosquito, thus eliminating the mosquito population as well as the parasite [16].

**Fig. 1:**
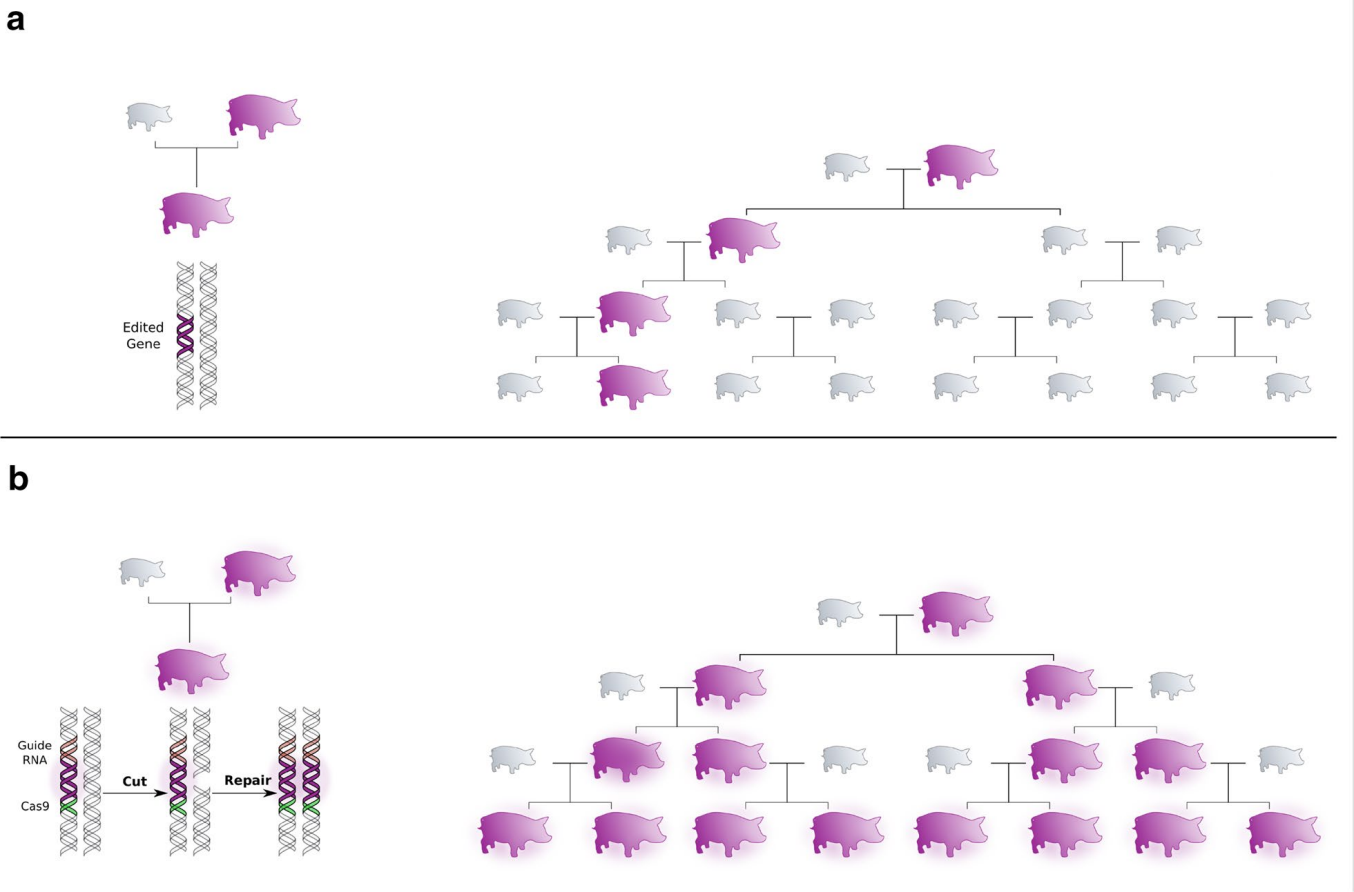
**a** Inheritance with genome editing and **b** inheritance with genome editing with gene drives

Gene drives could be combined with genome editing for quantitative traits to fix edited alleles more quickly in livestock populations. Each edited allele could have a gene drive based on a CRISPR/Cas9 editor. As shown in Fig. 1, the gene drive would be co-inherited with the edited allele across generations. This would ensure complete homozygosity for the favourable allele amongst all descendants of an edited individual, regardless of the genotype of the other parent.

The objective of this study was to quantify the potential of using gene drives with genome editing to increase the genetic gain for quantitative traits in livestock breeding.

## Methods

Simulation was used to evaluate the use of gene drives with genome editing in increasing the genetic gain for quantitative traits in livestock breeding. A variety of scenarios were tested, each using different editing strategies within the breeding program. All scenarios followed a common overall structure, where the simulation scheme was divided into historical and future components. The historical component was split into two parts: (1) evolution under the assumption that livestock populations have been evolving for tens of thousands of years prior to domestication; and (2) 21 recent generations of modern animal breeding with selection based on breeding values. The future component consisted of a further 20 generations of modern animal breeding. In each generation, parents of the next generation were selected based on true breeding values (TBV). Within a given scenario, the breeder was given the choice of using only selection, selection and genome editing, or selection and genome editing with gene drives. Recent historical animal breeding generations were denoted −20 to 0 and future animal breeding generations were denoted 1 to 20.

The simulations were designed to: (1) generate whole-genome sequence data; (2) generate quantitative trait nucleotides (QTN) affecting phenotypes; (3) generate pedigree structures for a typical livestock population; (4) perform selection; and (5) perform genome editing with and without gene drives. For each scenario, the genetic gain, genetic variance $(\sigma_{A}^{2} )$, rate of change in inbreeding ($\Delta F$), number of distinct QTN edited, rate of increase in favourable allele frequencies of edited QTN and the time to fix favourable alleles were evaluated. Results are presented as the mean of ten replicates for each scenario on a per generation and/or cumulative basis.

### Whole-genome sequence data, historical evolution

Sequence data was generated using the Markovian Coalescent Simulator (MaCS) [17] and AlphaSim [18, 19] for 1000 base haplotypes for each of ten chromosomes. Chromosomes were each 1 Morgan long comprising 10^8^ base pairs and were simulated using a per site mutation rate of 2.5 × 10^−8^, a per site recombination rate of 1.0 × 10^−8^ and an effective population size (N_e_) that varied over time in accordance with estimates for the Holstein cattle population [20]. N_e_ was set to 100 in the final generation of the coalescent simulation, to N_e_ = 1256, 1000 years ago, to N_e_ = 4350, 10,000 years ago, and to N_e_ = 43,500, 100,000 years ago, with linear changes in between these time-points. The resulting sequence had approximately 650,000 segregating sites in total.

### Quantitative trait variants

A quantitative trait was simulated by randomly sampling 10,000 QTN from the segregating sequence sites in the base population, with the restriction that 1000 QTN were sampled from each of the ten chromosomes. QTN had their allele substitution effect randomly sampled from a normal distribution with a mean of 0 and standard deviation of 0.01 (1.0 divided by the square root of the number of QTN). QTN effects were used to compute TBV for an individual.

### Pedigree structure, gamete inheritance and selection strategies

A pedigree of 41 generations of 1000 individuals in equal sex ratio was simulated. In the first generation of the recent historical animal breeding population (denoted generation −20), individuals had their chromosomes sampled from the 1000 base haplotypes. In each subsequent generation (i.e., generations −19 to 20), the chromosomes of each individual were sampled from parental chromosomes by recombination. A recombination rate of 1 Morgan per chromosome was simulated, resulting in a 10-Morgan genome. Recombination locations were simulated ignoring interference. In each generation, 25 males were selected to become the sires of the next generation using truncation selection on TBV. No selection was performed on females, and all 500 were used as dams.

### Genetic gain

Genetic gain was calculated in units of the standard deviation of TBV in the base generation (generation −20 or generation 1) as $\left( {\overline{{TBV_{curr} }} - \overline{{TBV_{base} }} } \right)/\sigma_{{TBV_{base} }}$, where $\overline{{TBV_{curr} }}$ is the mean TBV of the current generation and $\overline{{TBV_{base} }}$ and $\sigma_{{TBV_{base} }}$ are the mean and standard deviation of TBV in the base generation, respectively. Generation −20 was used as the base generation in order to observe the genetic improvement since the start of the recent historical breeding. Generation 1 was used for ease of presentation of some of the results. The genetic variance in each generation was calculated as: $\sigma_{A}^{2} = a^{\prime}a/\left( {n - 1} \right)$, where $a$ is a zero mean vector of TBV of the $n$ individuals in that generation.

### Efficiency of turning genetic variation into genetic gain

The efficiency of turning genetic variation into genetic gain at set generations was calculated by relating average genetic gain per generation to the rate of change in inbreeding of the future breeding component. The rate of change in inbreeding, $\Delta F$, was estimated by fitting a linear regression model $\log \left( {1 {-} F_{t} } \right) = \mu -\Delta Fg_{t}$, which is a linearization of formula $\Delta F = \left( {F_{t} - F_{t - 1} } \right)/\left( {1 - F_{t - 1} } \right)$ [21] and where $g_{t}$ is the mean breeding value at generation $t$. The efficiency of turning genetic variation into genetic gain was then calculated as the ratio of the average genetic gain per generation to $\Delta F$ as $\left( {100 \times [(G_{g} - G_{0} )/g]} \right)/\Delta F$, where $G_{0}$ is generation $0$ and $G_{g}$ is generation $g$ of the future breeding component.

### Scenarios

Three main scenarios were simulated: (1) selection alone; (2) selection and genome editing; or (3) selection and genome editing with gene drives. When editing with gene drives, the probability of successfully incorporating a gene drive with an edited allele, i.e., the conversion efficacy of the gene drive mechanism, was modelled. Three conversion efficacies of 0.5, 0.75 and 1.0 were compared.

When applying genome editing, a maximum of 500 edits per generation were allowed. In each generation, 25 sires were selected based on TBV and then either all 25 were edited at 20 QTN each or the top 5 were edited for 100 QTN each. For each sire, the QTN with the largest effect (i.e., $\alpha$) on phenotype for which the sire was not already homozygous for the favourable allele was edited, assuming that QTN effects were a priori known.

Unless explicitly mentioned, all results showing the effect of gene drives were run with the gene drive conversion efficacy set to 1.00 (i.e., 100% efficacy).

## Results

This paper uses simulation to examine how gene drives enhance the benefit of genome editing in breeding programmes with selection. The results highlight seven ways in which gene drives enhance the benefits of genome editing. Gene drives: (1) amplify the increase in genetic gain brought about by genome editing; (2) amplify the rate of increase in the frequency of favourable alleles and reduce the time it takes to fix them; (3) enable more rapid targeting of QTN with lesser effect for genome editing; (4) distribute fixed editing resources across a larger number of distinct QTN across generations; (5) focus editing on a smaller number of QTN within a given generation; (6) reduce the level of inbreeding when editing a subset of the sires; and (7) increase the efficiency of converting genetic variation into genetic gain.

### Genetic gain

**Fig. 2:**
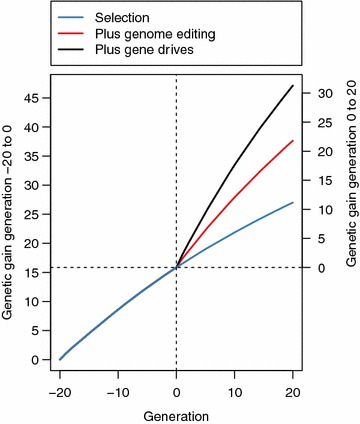
Genetic gain using selection (*blue line*), selection with standard genome editing (*red line*) or selection, genome editing with gene drives (*black line*). The figure represents the scenario when all 25 sires in a given generation were edited at 20 QTN each

Gene drives amplify the increase in genetic gain brought about by genome editing. This is shown in Fig. 2, which plots the overall genetic gain against time for generations −20 to 20 when all 25 sires were edited. Generations −20 to 0 were identical for all scenarios and represent the recent historical breeding, in which selection was used without editing. Generations 0 to 20 represented the future breeding where the breeder had the choice of using selection alone, selection and genome editing, or selection and genome editing with gene drives. Since generations −20 to 0 were identical for all scenarios and no editing was performed, all results presented are standardised to generation 0. Standardised genetic gain is given on the y-axis on the right in Fig. 2.

Figure 2 shows that by generation 20, gene drives achieved 1.43 times more genetic gain than genome editing [31.29 vs. 21.81; (see Additional file 1: Table S1)] and 2.80 times more genetic gain than selection alone [31.29 vs. 11.16; (see Additional file 1: Table S1)]. Genome editing achieved 1.95 times more genetic gain than selection alone [21.81 vs. 11.16; (see Additional file 1: Table S1)].

### Changes in allele frequency

**Fig. 3:**
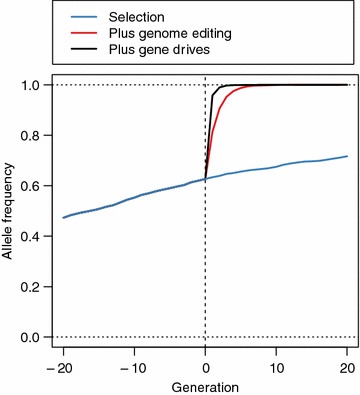
Allele frequency in each generation of the 20 QTN with the largest effect using selection (*blue line*), selection and genome editing (*red line*), or selection and genome editing with gene drives (*black line*). The figure represents the scenario when all 25 sires in a given generation were edited at 20 QTN each

Gene drives amplify the rate of increase in the frequency of favourable alleles at the QTN with the largest effect brought about by genome editing. This is shown in Fig. 3, which plots the average allele frequencies of the favourable alleles of the 20 QTN with the largest effect against time in generations −20 to 20. In the first generation of editing, gene drives produced nearly twice the increase in the frequency of favourable alleles at the 20 QTN with the largest effect than genome editing (increase of 0.33 vs. 0.18). This increase in frequency using gene drives was 47 times greater than that produced by selection alone, which produced an increase in the frequency of favourable alleles by 0.007 in the first generation and by 0.09 across all 20 generations.

Figure 3 also shows that gene drives fix the 20 QTN with the largest effect more quickly than genome editing alone. Gene drives achieve an asymptote of allele frequency higher than 0.99 in generation 2, whereas genome editing achieves it in generation 6. Selection without editing achieves a maximum frequency of approximately 0.72 across all 20 generations of future breeding. The rapid fixation of the 20 QTN with the largest effect when using gene drives would mean that QTN with lesser effect can be targeted for genome editing sooner.

**Fig. 4:**
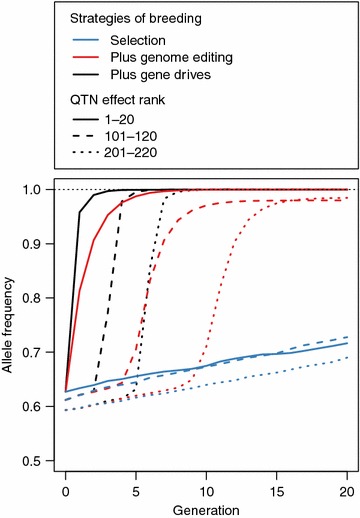
Allele frequency in the future 20 generations for QTN ranked by their effect (top 1 to 20, top 101 to 120, and top 201 to 220) using selection (*blue line*), selection and genome editing (*red line*), or selection and genome editing with gene drives (*black line*). The figure represents the scenario when all 25 sires in a given generation were edited at 20 QTN each. *Solid lines* represent the 1 to 20 QTN,* dashed lines* represent 101 to 120 QTN, and *dotted lines* represent 201 to 220 QTN

Gene drives reduce the time required to target QTN with lesser effect and increase the frequency of their favourable alleles more quickly. This is shown in Fig. 4, which plots the average allele frequencies of favourable alleles in three categories of QTN against time in generations −20 to 20. The three categories of QTN were: (1) the 20 QTN with the largest effects; (2) the 20 QTN with effect sizes ranked from 101 to 120; and (3) the 20 QTN with effect sizes ranked from 201 to 220.

Figure 4 shows that the slope of the lines for all three QTN categories are much steeper and occur at earlier generations when using gene drives. Selection without genome editing resulted in very small increases in allele frequencies for all three QTN categories across all 20 generations. Gene drives caused the shift in allele frequency to occur two times earlier than genome editing alone for both QTN category (2) (generation 2 vs. 4) and QTN category (3) (generation 5 vs. 9). Gene drives also reduced the time taken to reach allele frequencies higher than 0.95 by a half for both QTN category (2) (two vs. four generations) and QTN category (3) (two vs. five generations). This reduction in the time required to shift allele frequencies when using gene drives could have additional benefits in the maintenance and fixation of favourable alleles.

Figure 4 also shows that gene drives can result in the rapid fixation of favourable alleles at QTN with lesser effect, which would probably never become fixed and may even be lost using selection or genome editing alone. When using gene drives, an asymptote of average allele frequency higher than 0.99 was achieved for QTN categories (1), (2) and (3) in generations 2, 5 and 8, respectively. When using genome editing alone, this asymptote was reached only for category (1) while categories (2) and (3) had an asymptote of 0.98, which was reached in generations 14 and 17, respectively. This asymptote of 0.98 was caused by a loss of an average of four favourable alleles from the population before they could be targeted for genome editing. When using only selection, the maximum allele frequency reached for any category of QTN was approximately 0.72.

### Effect of gene drive efficacy on genetic gain

**Fig. 5:**
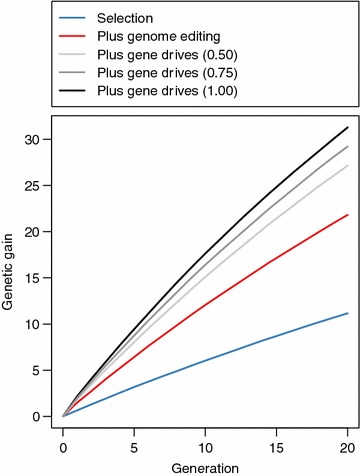
Genetic gain using selection (*blue line*), selection and genome editing (*red line*), or selection and genome editing with gene drives with conversion efficacies of 0.50 (*light grey line*), 0.75 (*grey line*), and 1.00 (*black line*). The figure represents the scenario where all 25 sires in a given generation were edited at 20 QTN each

Reducing the conversion efficacy of the gene drive mechanism reduces the genetic gain. This is shown in Fig. 5, which is a plot of the genetic gain against time in generations 0 to 20. At the three gene drive conversion efficacies that we tested, 1.00, 0.75 and 0.50, genetic gain was monotonically related to conversion efficacy. Gene drives with complete efficacy resulted in 1.07 times more genetic gain than gene drives with a conversion efficacy of 0.75 (31.29 vs. 29.22), and 1.15 times more genetic gain than gene drives with a conversion efficacy of 0.50 (31.29 vs. 27.16).

Gene drives with low conversion efficacies still substantially amplify the increase in genetic gain brought about by genome editing. Gene drives with a conversion efficacy of 0.50 resulted in 1.25 times more genetic gain than genome editing alone (27.16 vs. 21.81) and 2.43 times more genetic gain than selection alone (27.16 vs. 11.16).

### Focusing editing resources on a subset of sires: genetic gain

**Fig. 6:**
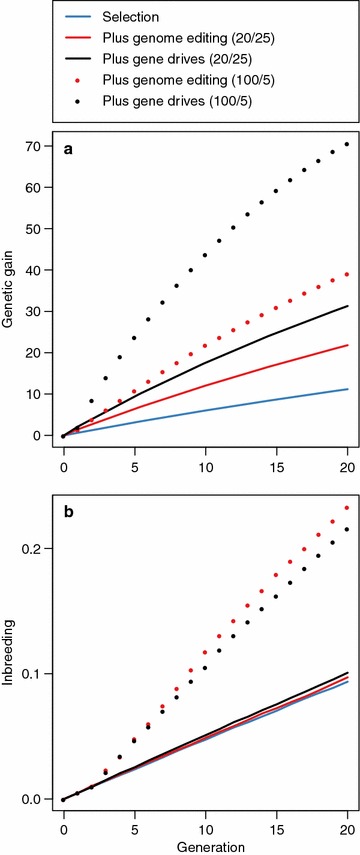
**a** Genetic gain and **b** inbreeding using selection (*blue line*), selection and genome editing (*red line*), or selection and genome editing with gene drives (*black line*) when either all 25 sires in a given generation were edited at 20 QTN (*solid lines*) each or the top 5 sires were edited at 100 QTN each (*dotted lines*)

Genetic gain was higher when editing a subset of the sires than when editing all 25 sires. This is shown in Fig. 6a, which plots the genetic gain against time in generations 0 to 20. Figure 6a shows scenarios in which either all 25 sires were edited at 20 QTN or the top 5 sires were edited at 100 QTN (both scenarios performed a total of 500 edits per generation).

Figure 6a shows that editing the top 5 sires resulted in more genetic gain than editing all 25 sires. This was the case with and without gene drives. With gene drives, editing the top 5 sires resulted in 2.25 times more genetic gain than editing all 25 sires (70.66 vs. 31.29). With genome editing alone, editing the top 5 sires resulted in 1.80 times more genetic gain than editing all 25 sires (39.17 vs. 21.81).

Figure 6a also shows that gene drives when editing the top 5 sires was the best strategy for maximising the genetic gain achieved. The lowest genetic gain was achieved when using selection alone. Editing the top 5 sires with gene drives resulted in 1.80 times more genetic gain than genome editing (70.66 vs. 39.17) and 6.33 times more genetic gain than selection alone (70.66 vs. 11.16). The second highest increase in genetic gain was achieved when editing the top 5 sires without gene drives. Editing the top 5 sires without gene drives resulted in 3.51 times more genetic gain than selection alone (39.17 vs. 11.16).

### Focusing editing resources on a subset of sires: inbreeding

Inbreeding levels were higher when editing a subset of the sires than when editing all 25 sires. This is shown in Fig. 6b, which plots the genetic gain against time in generations 0 to 20. Figure 6b shows scenarios in which either all 25 sires were edited at 20 QTN or the top 5 sires were edited at 100 QTN (i.e., both scenarios performed a total of 500 edits per generation). Editing the top 5 sires doubled the final maximum level of inbreeding observed with selection alone and when editing all 25 sires (~0.23 vs. ~0.10). The maximum level of inbreeding observed with selection alone and when editing all 25 sires was reached in half the time when editing the top 5 sires (generation 10 vs. generation 20).

Figure 6b also shows that the level of inbreeding attained when editing the top 5 sires was lower when gene drives were included. Figure 6b shows that at later generations, the reduction in inbreeding achieved with gene drives when editing the top 5 sires was more pronounced than in earlier generations. When editing the top 5 sires, the level of inbreeding attained with and without gene drives was equal across generations 0 to 5. By generation 20, the level of inbreeding reached without gene drives was 1.05 times higher than editing with gene drives (0.23 vs. 0.22).

### Efficiency of converting genetic variation into genetic gain

**Fig. 7:**
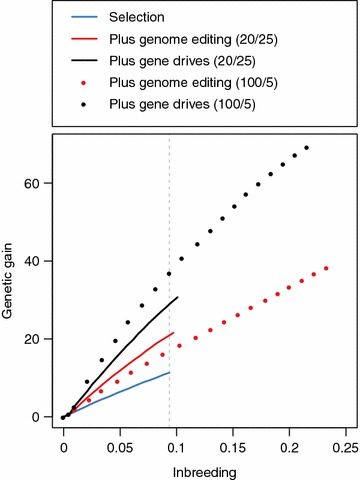
Genetic gain per change of inbreeding across the 20 generations of future breeding using selection (*blue line*), selection and genome editing (*red line*), or selection and genome editing with gene drives (*black line*). The figure represents the scenarios when either all 25 sires in a given generation were edited at 20 QTN (*solid lines*) each or the top 5 sires were edited at 100 QTN each (*dotted lines*)

Gene drives increase the efficiency of genome editing at converting genetic variation (measured by inbreeding) into genetic gain. This is shown in Fig. 7, which is a plot of the genetic gain against the inbreeding for generations 0 to 20. Figure 7 shows scenarios in which either all 25 sires were edited at 20 QTN or the top 5 sires were edited at 100 QTN (i.e., both scenarios performed a total of 500 edits per generation).

The two most efficient strategies were those including gene drives. The most efficient strategy was when the top 5 sires were edited with gene drives. The second most efficient strategy was when all 25 sires were edited with gene drives. The least efficient strategy was selection alone. By generation 20, the maximum level of inbreeding attained using selection alone was 0.0936 (indicated by the grey dashed vertical line in Fig. 7). At this level of inbreeding, editing the top 5 sires with gene drives achieved 3.92 times more genetic gain than selection alone (43.79 vs. 11.16). Editing all 25 sires with gene drives achieved 2.80 times more genetic gain than selection (31.29 vs. 11.16). Editing all 25 sires without gene drives achieved 1.95 times more genetic gain than selection alone (21.81 vs. 11.16). Editing the top 5 sires without gene drives achieved 1.78 times more genetic gain than selection alone (19.82 vs. 11.16).

The number of sires edited influences efficiency differently, depending on whether or not gene drives were incorporated. With gene drives, editing the top 5 sires was more efficient than editing all 25 sires. Without gene drives, editing all 25 sires was more efficient than editing the top 5 sires. At the maximum level of inbreeding attained using selection alone, editing the top 5 sires with gene drives resulted in 1.40 times more genetic gain than editing all 25 sires with gene drives (43.79 vs. 31.29). In comparison, editing all 25 sires without gene drives resulted in 1.10 times more genetic gain than editing the top 5 sires with gene drives (21.81 vs. 19.82).

The efficiency of turning genetic variation into genetic gain was higher when inbreeding was lower (i.e., in early generations) compared to when inbreeding was higher (i.e., later generations). This pattern was consistent across all scenarios. This is shown in Table 1 as the ratio between the genetic gain and the change in inbreeding from generation 0 to generations 4, 8, 12, 16 and 20.

**Table 1:** Efficiency of turning genetic variation into genetic gain in generations 4, 8, 12, 16, and 20 of future breeding

Editing strategy	Number of edited sires	Efficiency of turning genetic variation into genetic gain (95% CI)				
		Gen 4	Gen 8	Gen 12	Gen 16	Gen 20
Selection	0	1.34 (1.24–1.43)	1.27 (1.21–1.32)	1.21 (1.16–1.27)	1.18 (1.14–1.22)	1.14 (1.10–1.17)
Genome editing alone	25	2.65 (2.42–2.87)	2.51 (2.39–2.62)	2.38 (2.25–2.51)	2.27 (2.15–2.38)	2.16 (2.07–2.25)
With gene drives	25	3.66 (3.34–3.99)	3.43 (3.26–3.61)	3.25 (3.14–3.36)	3.10 (2.98–3.22)	2.95 (2.85–3.05)
Genome editing alone	5	2.12 (1.98–2.27)	1.73 (1.65–1.81)	1.52 (1.44–1.61)	1.44 (1.35–1.52)	1.38 (1.30–1.45)
With gene drives	5	4.83 (4.29–5.37)	3.84 (3.59–4.09)	3.38 (3.10–3.67)	3.06 (2.83–3.28)	2.76 (2.59–2.93)

*CI* confidence interval, *Gen* generation

For selection, the efficiency of turning genetic variation into genetic gain in generation 4 was 1.18 times higher than the efficiency in generation 20 (1.34 vs. 1.14). For genome editing, the efficiency in generation 4 was 1.23 times higher than the efficiency in generation 20 when all 25 sires were edited (2.65 vs. 2.16), and 1.54 times higher when the top 5 sires were edited (2.12 vs. 1.38). For genome editing with gene drives, the efficiency in generation 4 was 1.24 times higher than the efficiency in generation 20 when all 25 sires were edited (3.66 vs. 2.95), and 1.75 times higher when the top 5 sires were edited (4.83 vs. 2.76).

The reduction in efficiency across generations was greater when using gene drives than without. Table 1 shows that the decay in efficiency from generation 4 to 20 was larger when using gene drives than without. When editing all 25 sires with gene drives, the reduction in efficiency was 3.55 times greater than with selection alone (0.71 vs. 0.20). When editing the top 5 sires with gene drives, the reduction in efficiency was 10.35 times greater than with selection alone (2.07 vs. 0.20). Despite this, the use of gene drives was always more efficient than not using gene drives for all five generations tested.

### Effect of gene drives on the number of distinct QTN edited

Across all generations, gene drives enable the editing of a larger number of distinct QTN. This is shown in Table 2, which gives the average number of distinct QTN edited across the 20 future generations. The table gives all scenarios when either all 25 sires were edited at 20 QTN or the top 5 sires were edited at 100 QTN (i.e., both scenarios performed a total of 10,000 edits across all 20 generations). Table 2 shows that when editing all 25 sires, gene drives resulted in 1.89 times more distinct QTN being edited than with genome editing alone (656.3 vs. 346.7). When editing the top 5 sires, gene drives resulted in 2.21 times more distinct QTN being edited than with genome editing alone (2612.9 vs. 1179.7).

**Table 2:** Average number of distinct QTN edited across all 20 generations of future breeding using genome editing alone or with gene drives of different conversion efficacies

Gene drive conversion efficiency	Average number of distinct QTN edited (95% CI)	
	25 Sires edited at 20 QTN each	5 Sires edited at 100 QTN each
Genome editing alone	346.7 (340.4–353.0)	1179.7 (1168.6–1190.8)
0.50	508.2 (498.2–518.2)	1943.5 (1923.7–1963.3)
0.75	582.4 (576.9–587.9)	2264.9 (2244.6–2285.2)
1.00	656.3 (648.7–663.9)	2612.9 (2593.1–2632.7)

*CI* confidence interval

While the use of gene drives enabled the targeting of more QTN for editing across all 20 generations, the rapid fixation of favourable alleles means that within a given generation, gene drives focus the editing resources on a smaller number of QTN. This is shown in Table 3, which gives the average number of distinct QTN edited per generation. The table gives all scenarios when either all 25 sires were edited at 20 QTN or the top 5 sires were edited at 100 QTN (i.e., both scenarios performed a total of 500 edits per generation).

**Table 3:** Average number of distinct QTN edited per generation using genome editing alone or with gene drives of different conversion efficacies

Gene drive conversion efficiency	Average number of distinct QTN edited (95% CI)	
	25 Sires edited at 20 QTN each	5 Sires edited at 100 QTN each
Genome editing alone	61.1 (58.6–63.6)	203.0 (198.1–207.9)
0.50	60.0 (58.2–61.9)	184.2 (181.2–187.2)
0.75	59.9 (57.2–62.5)	180.2 (174.3–186.2)
1.00	59.1 (57.3–61.0)	172.8 (168.7–177.0)

*CI* confidence interval

Gene drives resulted in fewer distinct QTN edited per generation. This pattern was consistent when editing either all 25 sires or the top 5 sires. When editing all 25 sires, genome editing alone resulted in 1.03 times more distinct QTN being edited than with gene drives (61.1 vs. 59.1). When editing the top 5 sires, genome editing alone resulted in 1.17 times more distinct QTN being edited than with gene drives (203.0 vs. 172.8).

## Discussion

Our results highlight four main points for discussion, specifically: (1) the benefits of gene drives; (2) gene drives and editing strategies in livestock breeding; (3) gene drive risks and management strategies in livestock breeding; and (4) the assumptions made by the study and their effects on the application of gene drives in different settings.

### Benefits of gene drives

Our simulations show that gene drives could amplify the benefits of genome editing in livestock breeding. The main benefit of genome editing is that it increases short-, medium- and long-term genetic gain [1]. This increase is brought about by: (1) increasing the frequency of favourable alleles at QTN; (2) reducing the time to fix favourable alleles at the largest effect QTN; and (3) minimising the chance of loss of favourable alleles at QTN with lesser effect by genetic drift.

Although genome editing alone results in large increases in genetic gain, the time taken to fix favourable alleles at the QTN with the largest effect could be up to six generations ([1] and our results). This reduces the chance of fixing the favourable alleles of QTN with lesser effect, since they may never become targets for genome editing or the favourable allele may be lost by genetic drift before it becomes a target for editing or for selection.

For livestock species with large generation intervals, the six generations would mean that fixing the favourable alleles at only the QTN with the largest effect could require a decade or more. Fixing only these QTN with large effect may not be enough for the return on investment if most of the traits that form parts of breeding goals are quantitative and are influenced by many QTN, all with small effect.

Gene drives can overcome these limitations by reducing the time to fix favourable alleles at the QTN with the largest effect. This enables the targeting of QTN with lesser effect for editing at earlier generations. This means that favourable alleles at QTN with lesser effect can be maintained in the population, are less prone to loss by genetic drift and are much more likely to reach fixation within a shorter time frame. Our simulations show that gene drives can achieve 1.5 times the genetic gain achieved with genome editing and can achieve 3 times that achieved with selection.

### Gene drives and editing strategies in livestock breeding

With advances in genome editing technologies, genome editing of major genes within livestock breeding is a reality. More than 300 edits have been reported in livestock and plant species in the past five years, including edits for “double muscling” in pigs, cattle and sheep [4], to confer resistance to porcine reproductive and respiratory syndrome virus (PRRS) and African swine fever virus (ASFV) in pigs [4, 6–8], and has recently been adapted for use in poultry [22].

In spite of these advances, the economic and practical implications of genome editing means that it is likely that editing will be restricted to individuals with the largest impact on the population. In species such as pigs and cattle, these are the best performing males that are chosen as sires for the next generation. Editing these sires ensures that they are homozygous for the favourable allele. However, Mendelian sampling of alleles of the unedited dams means that there is no guarantee that all the progeny of an edited individual will also be homozygous for the favourable allele.

Gene drives eliminate the effect of Mendelian sampling by ensuring that all the offspring of an edited individual will be homozygous for the favourable allele, regardless of the genotype of its dam. In addition, all offspring will be homozygous for the gene drive, thus ensuring homozygosity in all future descendants of an edited individual [9, 10].

The economic and practical feasibility of genome editing may mean that the breeder must further prioritise amongst the selected sires. In this context, prioritising the top best performing sires for editing is the best option, and can even result in larger genetic gains over editing all sires. This increase in gain by editing only the best sires can be amplified by gene drives. We show that editing the top 5 best performing out of the 25 selected sires with gene drives can achieve over 6 times more genetic gain than selection alone and 2 times more genetic gain than editing the top 5 sires without gene drives.

The higher genetic gain achieved when editing a subset of the sires in this study is likely caused by the assumption of a fixed number of edits in a given generation (i.e., 500 edits per generation). This assumption meant that, within a given generation, a larger number of edits can be performed on a given individual when editing a subset of the sires than when editing all sires (i.e., top 5 sires edited at 100 QTN or all 25 sires edited at 20 QTN).

Applying a larger number of edits per individual in a subset of the sires means that the offspring of the edited subset perform better than the offspring of unedited sires and thus are more likely to be selected as parents for the next generation. The benefit of this is that the increase in frequency of favourable alleles occurs more quickly and results in higher genetic gains. The consequence of editing only a subset of the sires is that the increase in genetic gain comes at the expense of an increased rate of inbreeding.

Although gene drives cannot eliminate the increase in inbreeding observed when editing a subset of the sires, they can reduce it. They do this by speeding up the rate of spread of the favourable allele in the population (by implicitly editing the genome of non-edited mates of edited sires on the formation of zygotes). This achieves faster uniformity in performance across all individuals and reduces the relative advantage of the progeny and descendants of edited individuals both within and across generations.

Furthermore, gene drives increase the efficiency of converting genetic variation into genetic gain. This means that, for a given level of inbreeding, breeders could achieve more genetic gain with gene drives than with genome editing or genome selection alone. We show that when using gene drives, breeding programs can be four times more efficient than using selection alone and more than two times more efficient than using genome editing alone.

### Gene drive risks and management strategies in livestock breeding

The use of gene drives when editing livestock populations is novel and thus care should be taken to consider the potential risks involved in the design and use of such technology. The potential risks of gene drives in livestock breeding are: (1) incorrect identification of favourable alleles within a given generation; (2) accidental spread of gene drives from a farmed population to a natural population; and (3) mutation of gene drive elements. Careful use and design of gene drives could eliminate these risks. Although some of the necessary technologies and risk-alleviating techniques and strategies we mention below are in their infancy or not yet developed, the field of genome editing and gene drives is rapidly advancing, and we believe that such technologies will eventually be available. Once developed, these technologies will need to be tested both in silico and in vivo before they can be applied at a larger scale within livestock breeding programs.

#### Risk 1: incorrect identification of favourable allele

The gene drive mechanism is very powerful at quick dissemination of alleles through a population. If the alleles are favourable and remain favourable into the future, there would be no negative consequences. However, if the alleles are incorrectly identified as favourable due to bad allele choice driven by underpowered experiments and less dependable data, or become unfavourable due to a change in environment, breeding goals or changes in the genetic background (e.g., negative epistasis), the rapid spread of a particular allele through a population could be negative or even catastrophic [23].

To overcome this, the gene drive mechanism could be used to switch back to the alternative allele in future generations. Alternatively, an additional gene drive could be introduced to deactivate and eliminate the initial gene drive from the genomes of future generations [12, 16, 24, 25]. This would be possible by combining the gene drive with a mechanism of underdominance, whereby individuals that are heterozygous for the deactivated gene drive would have a lower fitness than homozygous individuals [10, 26].

To further minimise the impact of incorrect allele identification, gene drives could be used to increase only the frequency of favourable alleles with proven effects. Those that appear to have favourable effects, but for which effects have not yet been proven, could be increased in frequency more conservatively using standard genome editing approaches.

#### Risk 2: accidental release of gene drives into wild populations

In livestock breeding schemes, the accidental introduction of the gene drive mechanism into a natural population could occur if a domesticated animal carrying a gene drive mates with an animal in a natural population from the same or related species. If an accidental introduction of the gene drive mechanism into a natural population did occur, it would result in the quick spread of the allele through the population. An allele that is considered favourable in farmed animals (e.g., double muscling) may be detrimental to the fitness of natural populations.

As a way of minimising this risk, physical containment strategies to reduce the likelihood of the gene drive escaping into natural populations could be used [24]. However, in some breeding programs physical containment may not be entirely possible. For example, the marine stage of the Atlantic salmon lifecycle in a breeding system takes place in seawater cages, where the possibility of escape and breeding with natural populations is quite high.

In such cases, alternative biological ways could be used to contain the gene drive system. These could involve attaching elements to the gene drive mechanism that control the number of times that the gene drive mechanism could act. A hypothetical example of this could involve adding five such elements and that each time the gene drive mechanism worked one of these elements was lost. Thus, the gene drive mechanism would only remain active in five descendant generations. To our knowledge no such mechanism has been developed, but the recently proposed ‘daisy drive system’ [27] bears some resemblance. The daisy drive system is identical in its effect to the normal gene drive system, but differs in its design. It involves a series of $n$ unlinked gene drive elements that are unable to drive the spread of their own allele, but that control the spread of the gene drive element above it in the chain. Our results suggest that a gene drive mechanism with an element that enabled it to act for only two or three generations would convey all of the benefits of the efficiency of gene drives while removing the element of risk.

#### Risk 3: mutation of gene drive elements

Gene drives have two major components: (1) a guide RNA, which is the part of the gene editing mechanism used to recognise the specific target region of the genome where the gene drive will be incorporated; and (2) the *Cas9* gene, whose protein product is responsible for cleavage of the targeted genomic region in order to initiate DNA repair. Without the *Cas9* gene, the gene drive mechanism is non-functional [28].

If the guide RNA mutates and is no longer able to specifically recognise the original targeted region, the gene drive mechanism could be incorporated into off-target regions of the genome. This would result in the uncontrolled and rapid spread of alleles at off-target regions with unknown consequences. Careful design of guide RNAs that require multiple mutations in order to target different genomic regions would minimise the probability of off-target incorporation of gene drives in future generations [24]. Alternatively, the guide RNA and the *Cas9* gene could be partitioned into separate cassettes. The genomic locations of the two cassettes could be carefully designed so that initial linkage between them ensures co-inheritance [15, 24, 29]. Recombination over a number of generations would break up this initial linkage, thus inactivating the gene drive mechanism in individuals who inherit only one of the cassettes and bypassing the problem of deleterious mutations accumulating in the gene drive over time.

### Assumptions and applications

The benefits of gene drives are applicable in the context of some assumptions made in this study that are patently over simplified and technologically not possible currently. These include the genetic architecture of the trait of interest and the ability to discover many causal variants for quantitative traits, the absence of dominance, pleiotropy and epistasis, the ability to perform multiplexed genome editing, not accounting for the costs associated with each edit, and the absence of certainty that gene drives can be safely used (as discussed above in the section: “Gene drive risks and management strategies in livestock breeding”). We believe that the advances in genome sciences that will be made in the next decade or so will help to provide solutions to these simplifications, and provide some discussion around these assumptions below.

### The impact of trait architecture and the discovery of causal variants on including gene drives

Potential targets for genome editing are already available in a variety of species for qualitative traits, but this is not always true for quantitative traits. The majority of traits forming breeding goals in livestock are quantitative, therefore it will be necessary to identify good targets in order to maximise the potential of this technology in livestock breeding. We chose to evaluate gene drives for a quantitative trait under the assumption that targets for editing were known and that the inheritance of the trait was additive.

In this study, QTN were prioritized for editing based on effect size. We do not believe that modest errors in the ordering of QTN would alter the results. Rather we believe that to use this technology, a breeding program would need to be able to find the approximately 500 to 600 of the QTN with the largest effect that control the genetic variation of the trait or selection index at some point over a 20-year time period.

We show that large positive impacts on genetic gain can be achieved with as little as 20 targets with large effect in any given generation. The identification of 20 or 30 targets for editing in the next few years is likely to be possible within large breeding schemes that routinely record and collect dense phenotypic and genomic data. However, the total number of targets that would need to be identified over several years is actually rather large (e.g., 500 to 600) and is more challenging. Our simulations show that these QTN would have to be discovered over a 20-year period. This may be possible given the huge advances in genome science that have been made in recent years and are likely to be made in the next two decades. Many breeding schemes are moving towards routine collection of sequence data, which will help in the precise identification and mapping of more QTN with large effect to target. Explicit approaches to discover genome editing targets will be needed. These approaches could make use of many different technologies including sequence enabled genome-wide association studies, genome annotation data, gene expression data, genome editing in vivo and in vitro and matings that are explicitly designed to enable allele-testing [30].

### Impact of dominance, pleiotropy and epistasis on including gene drives

We also assume that the inheritance of the quantitative trait is fully additive. However, dominance and epistatic effects may exist, and could influence the number of edits required for a given individual and for a given QTN within a generation. For example, dominance of the favourable allele would mean that frequencies of favourable alleles need only be increased to ensure that individuals carry a minimum of one copy of the favourable allele, which would require fewer edits for a given QTN and may be done without the inclusion of gene drives.

In this study, a single trait controlled by 10,000 QTN each with additive effects sampled from a Gaussian distribution was simulated. This is a simplification, since most livestock breeding programs select for multiple traits. These traits have complex correlations with each other, caused by pleiotropy and linkage between alleles at QTN that affect different traits. However, we do not believe that the main conclusions from our results would be very different, since most livestock breeding programs select on an index. This index behaves like a single trait that is affected by many loci and thus our single-trait model could be seen as implicitly accounting for pleiotropic effects and complex genetic correlations between the component traits.

Negative epistasis of QTN may mean that editing multiple QTN for a given individual is required. We did not simulate epistasis because the data and theory suggest that epistasis has a minor contribution to total variation [31, 32]. However, if there are large epistatic effects, the value of genome editing and gene drives in livestock breeding would be significantly reduced. This is because on the one hand, the frequency of individual alleles would be shifted very rapidly by genome editing, resulting in these alleles being placed in different haplotypes that could have very different effects. On the other hand, this would also reveal epistatic effects that might otherwise be difficult to observe due to limited recombinations. The impact of epistasis is an open question that needs to be addressed with real data and populations.

### Impact of multiplexed genome editing on including gene drives

The results of this study imply that multiplex editing of many alleles is needed to generate large increases in genetic gain in livestock. To our knowledge large multiplexing (e.g., 10 or more alleles) has not been successfully performed to date. However, genome editing techniques are improving rapidly and are an intensive area of research across all of the life sciences. We anticipate that multiplex genome editing will be possible in the future.

### Impact of cost on including gene drives

The cost assumption made in this study was that a fixed editing resource of 500 edits was available within a given generation. These 500 edits could be distributed so that either all 25 selected sires were edited at 20 QTN each, or the top 5 sires were edited at 100 QTN each. If the cost of editing an individual is high, editing more QTN per individual enables a faster spread of favourable alleles across the population. In this context, gene drives will increase the rate of spread of favourable alleles throughout the population and reduce the impact of inbreeding. If the cost of a single edit is high, gene drives will be even more important for the fast dissemination of favourable alleles into the population. This is because within livestock breeding schemes where the majority of individuals are descendants of a few sires, editing with gene drives will mean that descendants of edited individuals will never require editing. Therefore the number of edits required in future generations for a given QTN is minimised.

Another assumption made was that gene drives do not constitute an additional edit by themselves. With the rapid fixation of the QTN with the largest effect with gene drives, this assumption meant that additional edits were available for QTN with lesser effect in future generations. If the gene drive is counted as an additional edit or if the cost of gene drives is too high, individual cost-benefit analyses would need to be conducted to evaluate the benefits of gene drives in the context of population size and structure and trait architecture.

## Conclusions

Genome editing in livestock could be used to increase the frequency of favourable alleles at QTN with large effect. Gene drives could be used to increase the speed at which edited alleles are spread across livestock populations. They would do this by eliminating the effect of Mendelian sampling of alleles in unedited mates, resulting in complete homozygosity for the favourable allele amongst all descendants of an edited individual. Faster fixation of favourable alleles would mean that fewer edited founders would be required to fix the favourable allele for a given QTN. This would enable the targeting of more QTN and more effective distribution of editing resources across generations. Faster fixation of favourable alleles would also result in larger genetic gains in shorter time spans. In our simulations, we show that the larger genetic gains would come at no expense in inbreeding. In fact, gene drives could reduce the levels of inbreeding and increase the efficiency of the breeding program by resulting in higher genetic gains for a given unit of inbreeding. The magnitude of the benefits of gene drives would depend on three main factors: (1) the genetic architecture of the trait of interest, (2) the associated costs, and (3) risk management. Therefore, the additional benefits achieved with gene drives should be evaluated within individual breeding programs by conducting tailored cost-benefit analyses, taking into account population size and structure, trait architecture and the ability to successfully control the power of the technology.

## Additional file

**Additional file 1: Table S1.** Fold increase in genetic gain achieved using genome editing with gene drives when standardised to generation 0. The table demonstrates the fold increase when all 25 sires in a given generation were edited at 20 QTN.
