# Supplementary material for: Potential of gene drives with genome editing to increase genetic gain in livestock breeding programs
Source: Genet Sel Evol. 2017 Jan 4;49:3. doi: 10.1186/s12711-016-0280-3 (PMC5240390; doi:10.1186/s12711-016-0280-3)
Supplement: Supplementary file 1 — Additional file 1: Table S1. Fold increase in genetic gain achieved using genome editing with gene drives when standardised to generation 0. The table demonstrates the fold increase when all 25 sires in a given generation were edited at 20 QTN. [file 12711_2016_280_MOESM1_ESM.docx]

**Supplementary Material**

Supplementary Table 1 – Fold increase in genetic gain achieved using genome editing with gene drives when standardised to generation 0. The table demonstrates the fold increase when all 25 sires in a given generation were edited at 20 QTN.

|  | **Fold increase in genetic gain with gene drives** | |
| --- | --- | --- |
| **Gene drive conversion efficiency** | **Relative to selection alone** | **Relative to genome editing alone** |
| Genome editing only | 1.95  (1.93–1.98) | - |
| 0.50 | 2.43  (2.40–2.46) | 1.25  (1.23–1.26) |
| 0.75 | 2.62  (2.59–2.65) | 1.34  (1.33–1.35) |
| 1.00 | 2.80  (2.78–2.83) | 1.43  (1.42–1.45) |
